# A Protective HLA Extended Haplotype Outweighs the Major COVID-19 Risk Factor Inherited From Neanderthals in the Sardinian Population

**DOI:** 10.3389/fimmu.2022.891147

**Published:** 2022-04-19

**Authors:** Stefano Mocci, Roberto Littera, Stefania Tranquilli, Aldesia Provenzano, Alessia Mascia, Federica Cannas, Sara Lai, Erika Giuressi, Luchino Chessa, Goffredo Angioni, Marcello Campagna, Davide Firinu, Maria Del Zompo, Giorgio La Nasa, Andrea Perra, Sabrina Giglio

**Affiliations:** ^1^ Medical Genetics Unit, Department of Medical Sciences and Public Health, University of Cagliari, Cagliari, Italy; ^2^ Medical Genetics Unit, R. Binaghi Hospital, Local Public Health and Social Care Unit (ASSL) of Cagliari, Cagliari, Italy; ^3^ Association for the Advancement of Research on Transplantation O.d.V., Non Profit Organisation, Cagliari, Italy; ^4^ Medical Genetics Unit, Department of Experimental and Clinical Biomedical Sciences “Mario Serio”, University of Florence, Florence, Italy; ^5^ Department of Medical Sciences and Public Health, University of Cagliari, Cagliari, Italy; ^6^ Liver Unit, Department of Internal Medicine, University Hospital of Cagliari, Cagliari, Italy; ^7^ Structure of Infectious Diseases Unit, SS Trinità Hospital, Cagliari, Italy; ^8^ Department of Biomedical Sciences, University of Cagliari, Cagliari, Italy; ^9^ Hematology Unit, Businco Hospital, Department of Medical Sciences and Public Health, University of Cagliari, Cagliari, Italy; ^10^ Unit of Oncology and Molecular Pathology, Department of Biomedical Sciences, University of Cagliari, Monserrato, Italy; ^11^ Centre for Research University Services (CeSAR, Centro Servizi di Ateneo per la Ricerca), University of Cagliari, Monserrato, Italy

**Keywords:** COVID-19, SARS-CoV-2, Neanderthal Covid-19 risk haplotype, rs35044562 variant, HLA-A*02:01, B*18:01, DRB*03 extended haplotype

## Abstract

Sardinia has one of the lowest incidences of hospitalization and related mortality in Europe and yet a very high frequency of the Neanderthal risk locus variant on chromosome 3 (rs35044562), considered to be a major risk factor for a severe SARS-CoV-2 disease course. We evaluated 358 SARS-CoV-2 patients and 314 healthy Sardinian controls. One hundred and twenty patients were asymptomatic, 90 were pauci-symptomatic, 108 presented a moderate disease course and 40 were severely ill. All patients were analyzed for the Neanderthal-derived genetic variants reported as being protective (rs1156361) or causative (rs35044562) for severe illness. The β°39 C>T Thalassemia variant (rs11549407*)*, HLA haplotypes, KIR genes, KIRs and their HLA class I ligand combinations were also investigated. Our findings revealed an increased risk for severe disease in Sardinian patients carrying the rs35044562 high risk variant [OR 5.32 (95% CI 2.53 - 12.01), p = 0.000]. Conversely, the protective effect of the HLA-A*02:01, B*18:01, DRB*03:01 three-loci extended haplotype in the Sardinian population was shown to efficiently contrast the high risk of a severe and devastating outcome of the infection predicted for carriers of the Neanderthal locus [OR 15.47 (95% CI 5.8 – 41.0), *p* < 0.0001]. This result suggests that the balance between risk and protective immunogenetic factors plays an important role in the evolution of COVID-19. A better understanding of these mechanisms may well turn out to be the biggest advantage in the race for the development of more efficient drugs and vaccines.

## Introduction

Sardinia is the second largest island of the Mediterranean Sea and has always been considered an outlier in the genetic landscape of the European continent (1–3). The genetic structure of its population probably stems from the unprecedented influx of migrants arriving on the island from Southeastern Europe during the Neolithic Era (4, 5). This Neolithic founder population remained genetically isolated for a few thousand years from the later Bronze Age expansions occurring across Europe (6, 7).

Despite more than a century of linguistic and cultural Italianization and the more recent globalization, the unique genetic heritage of the inhabitants of Sardinia continues to survive today, as was witnessed once again during the Coronavirus disease 2019 (COVID-19) pandemic. Despite the emergence of different SARS-COV-2 variants, the Sardinian population continued to register one of the lowest rates of intensive care unit admissions and the lowest incidence of mortality with respect to the remaining Italian regions (8, 9).

It is well known that advanced age, male gender, obesity, diabetes, and other comorbidities (10, 11) are all well-established risk factors but are not sufficiently predictive of progression to the severe and often fatal forms of COVID-19 infection. This evidence suggests that among the factors that contribute to determining the severity of the disease, one of the main ones is certainly the role of genetic variability in individual susceptibility and response to viral infections (12–15).

Within this context, an important role is played by the human leucocyte antigen (HLA) class I and class II molecules which are critical components of the antigen presentation pathway involved in alerting the immune system of virally infected cells. A large variety of alleles and/or haplotypes have been found either associated with an increased risk for the more severe clinical manifestations of SARS-CoV-2 or capable of exerting a protective effect against the disease (16, 17). This multitude of studies is mainly the result of differences in the frequencies of HLA alleles and haplotypes observed across ethnicities (18). In Sardinia, for example, the HLA system exhibits a relatively low level of polymorphism, so much so that the ten most frequent HLA extended haplotypes correspond to approximately half of the haplotypes present in the entire population (19). One of these is represented by the extended HLA haplotype HLA A*02:05, B*58:01, C*07:01, DRB1*03:01 which seems to exert a protective effect against SARS-CoV-2 (12).

Further protection could derive from interactions between killer-cell immunoglobulin-like receptors (KIRs), expressed on the surface of natural killer (NK) cells and their cognate human leukocyte antigen (HLA) Class I ligands (20, 21). A recent study performed on the population of central southern Sardinia showed that *KIR2DS2* in combination with HLA-C alleles of the C1 group exerts a potent protective effect against the adverse outcomes of COVID-19 (22).

Other studies have recently produced evidence for association of a Neanderthal haplotype with an increased risk of severe COVID-19. Among 13 loci found to be associated with infection, the 3p21.31 locus conferred the highest risk for a serious outcome (23–25). This locus spans 49.4 thousand kilobases (kb) and contains several genetic variants located on the leucine zipper transcription factor-like 1 (*LZTFL1*) gene which are all in high linkage disequilibrium and constitute a specific haplotype, similar to the corresponding genomic region of the Vindija 33.19 Neanderthal (26, 27).

The Neanderthal haplotype (chr3:45,859,651-45,909,024, hg19), which is most strongly associated with the risk of developing a severe form of COVID-19, is present at a frequency of approximately 30% in South Asia and 8% in Europe and contains four SNPs (rs35044562, rs73064425, rs34326463, rs67959919) (26, 27).The significant differences observed for the carrier frequencies have previously been studied in Bangladesh where more than half the population (63%) carries at least one copy of the Neanderthal risk haplotype and a further 13% are homozygous as a consequence of positive selection (23, 28). It can therefore be estimated that individuals of Bangladeshi descent are at least twice as likely to die from COVID-19 in comparison to individuals of the general population lacking these Neanderthal-inherited gene segments (28).

Conversely, a Neanderthal haplotype of ∼75 kb on chromosome 12 (chr12: 113,350,796 to 113,425,679; hg19) has been reported as having a protective effect against the more severe forms of SARS-CoV-2 infection (29, 30).

This haplotype - which is fairly common in all world regions outside Africa - spans the *OAS1, OAS2*, and *OAS3* genes encoding oligoadenylate synthetases (29). The 2’-5’-oligoadenylate synthetase family of interferon-induced, double-stranded RNA-dependent enzymes is well known for its important role in immune-mediated host defense mechanisms against viral infections (29–32).

Given the numerous genetic peculiarities of the Sardinian population deriving from founder effects and genetic drift (33), we decided to search for protective factors capable of counteracting the high risk of developing severe COVID-19 exerted by the Neanderthal haplotype which could possibly explain the lower impact of the disease on the island during the pandemic.

We studied the frequency of rs35044562, which Zeberg et al. identified as the major risk allele (23), in both COVID-19 patients and the general population of Southern Sardinia. We also focused on the frequency of the rs1156361 allele, which seems to exert a protective effect against severe COVID-19 (29). Subsequently, we analyzed these Neanderthal polymorphisms in combination with a series of immunogenetic factors previously found to have a major influence on the clinical progression of SARS-CoV-2 infection in Sardinia: β°39 C>T thalassemia mutation (rs11549407*)*, *HLA* and *KIR* genes and combinations of KIRs and their HLA-C ligands (12, 22).

We demonstrated that the severe clinical course of COVID 19 infection predicted for Neanderthal haplotype carriers was effectively counteracted by the protective effect exerted by the three-locus extended haplotype HLA-A*02, B*18, DRB1*03, once again confirming the crucial role of HLA molecules in immune response mechanisms, including those responsible for the different infection rates of SARS-COV-2 variants.

## Materials and Methods

Patients were recruited and enrolled in the study at the Department of Medical Sciences and Public Health of the University of Cagliari, the University Hospital of Cagliari (AOUCA), and the SS. Trinità Hospital of the Sardinian Regional Company for the Protection of Health (ATS Sardegna).

Written informed consent was obtained prior to the study from all patients and controls in accordance with national and institutional ethical standards of the local human research committee and in conformity with the ethical guidelines of the Declaration of Helsinki. The study was approved by the responsible ethics committee (Ethics Committee of the Cagliari University Hospital; date of approval: May 27, 2020; protocol number GT/2020/10894). Records of written informed consent are kept on file and in the clinical record of each patient.

A total of 358 unvaccinated COVID-19 patients were recruited into the study over a period of 6 months starting from the month of June 2020. All patients were diagnosed with SARS-CoV-2 by reverse transcriptase-polymerase chain reaction (RT-PCR) from a nasopharyngeal swab and assigned to one of four groups based on disease severity. According to WHO classification, patients with severe disease are those who need invasive mechanical ventilation or high-flow nasal oxygen in a hospital setting, while those classified as having moderate symptoms do not require oxygen. Pauci-symptomatic patients only have mild symptoms such as fever, malaise, cough, sore throat, muscle pain, gastrointestinal complaints, loss of taste and/or smell but do not have breathing problems or abnormal results on chest imaging.

All 358 patients enrolled in the study, were evaluated according to the severity of the clinical manifestations: 120 were asymptomatic, 90 were pauci-symptomatic, 108 presented a moderate disease course and 40 were severely ill.

The relative genotype frequencies in the general population were studied on a cohort of 314 individuals who were selected from the Sardinian voluntary bone marrow donor register in order to avoid any possible correlation with SARS-CoV-2 infection. The control population adequately represented the male to female ratio and was taken from the same geographical areas in central southern Sardinia as the patients. Overall, it was considered to be highly representative of the genetically homogeneous insular population.

All 358 patients were investigated for the presence of the risk alleles at rs35044562, rs73064425, rs34326463, rs67959919 and the protective allele at rs1156361. Other immunogenetic factors previously found associated with SARS-CoV-2 infection in the Sardinia population were also investigated: β°39 C>T Thalassemia variant (rs11549407*)*, HLA haplotypes, KIR genes, KIRs and their HLA class I ligand combinations (12, 22).

### DNA Extraction and Quantification

Genomic DNA was extracted from EDTA anticoagulated blood using QIAamp DNA blood mini kit (Qiagen, Hilden, NW, Germany) according to the manufacturer’s instructions. Genomic DNA was eluted in 200 μl of Elution Buffer H_2_O. DNA was quantified using the Qubit 3.0 fluorimeter (Life Technologies, Carlsbad, California, USA), following the manufacturer’s instructions (Qubit dsDNA BR and Qubit dsDNA HS Assay Kits).

### DNA Amplification

Primer pairs for each region of interest were designed using Primer3 (34). The annealing temperature was optimized for each forward and reverse primer set. The four SNPs located within the *LZTFL1* gene (rs35044562, rs73064425, rs34326463, rs67959919*)*, one SNP on *OAS3* (rs1156361), and the rs11549407 within the *HBB* gene are reported in the **Supplementary Table S1**. The PCR reaction was performed according to the protocol supplied with AmpliTaq Gold™ DNA Polymerase (Applied Biosystems/Thermo Fisher Scientific, Waltham, MA).

### Sanger Sequencing

Sequencing was performed using the BigDye™ Terminator v3.1 Cycle Sequencing Kit (Applied Biosystems, USA), with the same primers described previously and cleaned up with CleanSEQ Dye-Terminator Removal Kit (Beckman Coulter, Inc.). Capillary electrophoresis was performed on the ABI 3500 Genetic Analyser (Applied Biosystems) and sequences were analyzed with Sequencher 5.3 (^©^ 2017 Gene Codes Corporation).

### HLA and KIR Genotyping

Class I and class II alleles were typed in patients and the control population using a next generation sequencing (NGS) platform. Class I (HLA-A,-B, and -C) and Class II (HLA-DRB1, HLA-DQA1, HLA-DQB1 and HLA-DPB1) genotyping was performed using commercially available NGS 7-Lociamplificationkit (OmixonHolotypeHLA™, For MiSeq Illumina), according to the protocol supplied by the manufacturer.

Genotype assignment was performed using HLATwinsoftware (Omixon, Inc). Any HLA alleles resulting to be rare or ambiguous were systematically re-typed according to the Sanger sequencing-based typing (SBT) method with the following kits: AlleleSEQR^®^HLA for the HLA-A, -B, -C and -DRB1 loci and SBTexcellerator^®^ for the HLA-DQA1, -DQB1 and -DPB1 loci (GenDx&GenDx Products, Utrecht, The Netherlands).

HLA-C alleles were assigned to the C1 or C2 ligand category by evaluating the dimorphism (asparagine or lysine) at position 80 of the HLA-C molecule. The HLA-B alleles were divided into 2 groups according to the expression of the HLA-Bw4 or HLA-Bw6 epitopes. The two isoforms of HLA-Bw4 were discriminated by the presence of leucine (Bw4Ile80) or threonine (Bw4Thr80) in position 80. HLA-A23, -A24, and -A32 pertain to the HLA-Bw4Ile80 group of serological epitopes (35).

The presence of the 14 KIR genes (*KIR2DL1, KIR2DL2, KIR2DL3, KIR2DL4, KIR2DL5, KIR3DL1, KIR3DL2, KIR3DL3, KIR2DS1, KIR2DS2, KIR2DS3, KIR2DS4, KIR2DS5* and *KIR3DS1*) was determined in patients and the control population using PCR-SSP with primers specific for each locus according to a previously reported method (36–39).

To confirm and validate our results, KIR gene typing was repeated on a total of 120 randomly selected samples among patients and controls using an alternative method: PCR-SSP (Olerup SSP^®^ KIR Genotyping). The concordance between the two methods was 99.8%.

### Statistical Analysis

Continuous variables were expressed as mean ± standard deviation. IBM SPSS Statistics for Windows, version 20.0 (IBM, Armonk, NY, USA) was used to calculate the odds ratios (ORs) and 95% confidence intervals (CIs). The χ2 test was used to compare the distribution of genotype and allele frequencies between the four different groups of COVID-19 patients and the population-control groups. Deviation from Hardy–Weinberg equilibrium was performed using Haploview 4.0 software (Broad Institute, Cambridge, MA, USA) (40). All tests were two-sided and only values of p < 0.05 were accepted as being statistically significant.

## Results

### Neanderthal-Derived Genetic Variant Frequencies in Sardinian COVID-19 Patients and Controls

We evaluated a total of 672 blood samples of the southern Sardinian population, corresponding to 358 COVID-19 cases (166 females and 192 males) and 314 individuals of the control population (139 females and 175 males). Patient mean age was 57.4 ± 16.9 years and the control population mean age was 45.5 ± 8.9 years. Four single nucleotide polymorphisms (SNPs) in strong linkage disequilibrium (rs35044562, rs73064425, rs34326463, rs67959919) were evaluated, considering rs35044562 as the index risk variant for severe COVID-19 infection. The index risk (rs35044562) and protective (rs1156361) Neanderthal variants revealed a distribution in Hardy–Weinberg equilibrium (HWE) both in SARS-CoV-2 patients and the control population. Indeed, the $X_{HWE}^{2}$ p-values for the rs35044562 and rs1156361 variants did not reach statistical significance in the control population ( $X_{HWE}^{2}$ = 0.82, *p* = 0.37and $X_{HWE}^{2}$ = 0.13, *p*= 0.72, respectively). Also, in the patient group, these two variants were confirmed to be in HWE ( $X_{HWE}^{2}$ = 0.57, *p*= 0.45and $X_{HWE}^{2}$ = 0.004, *p*= 0.95 respectively). The distribution of the allele and genotype frequencies are shown in **Table 1**.

**Table 1:** Allele and Genotype distribution of rs35044562 and rs1156361 in the control population and COVID-19 patients.

Allele and Genotype distribution						
Gene	SNP	Control population (n = 314)			COVID-19 patients (n = 358)	
*LZTFL1*	rs35044562 (A>G) (n = 672)	Allele	(%)	$X_{HWE}^{2}$ 0.82, *p* = 0.37	(%)	$X_{HWE}^{2}$ 0.57, *p*= 0.45
		A	87.7		90.5	
		G	12.3		9.5	
		Genotype	(%)		(%)	
		AA	76.4		81.6	
		AG	22.6		17.9	
		GG	1.0		0.5	
*OAS3*	rs1156361 (T>C) (n = 672)	Allele	(%)	$X_{HWE}^{2}$ 0.13, *p* = 0.72	(%)	$X_{HWE}^{2}$ 0.004, *p* = 0.95
		T	35		34.4	
		C	65		65.6	
		Genotype	(%)		(%)	
		TT	12.7		11.7	
		TC	44.6		45.3	
		CC	42.7		43.0	

$X_{HWE}^{2}$ =Hardy-Weinberg equilibrium Chi square value; p = Hardy-Weinberg equilibrium p value.

No significant differences were observed between patients and the control population for the A and G allele frequencies or the AA, AG and GG genotype frequencies of the rs35044562 high risk variant.

Similar results were obtained for the T and C allele frequencies and the TT, TC and CC genotype frequencies of the rs1156361 protective variant (**Table 1**).

### Correlation Between rs35044562 (*LZTFL1* Gene) and rs1156361 (*OAS3* Gene) and the Severity of Clinical Manifestations in SARS-CoV-2 Infection

The patients were then divided into two groups based on disease severity: Group 1 contained asymptomatic, pauci-symptomatic and moderately ill SARS-CoV-2 patients, whereas group 2 contained severely ill patients.

Patients with severe clinical manifestations had significantly higher allele and genotype (AG and GG) frequencies for the rs35044562/G variant compared to the group of asymptomatic, pauci-symptomatic and moderately ill SARS-CoV-2 patients (22.5% vs 7.9%; OR_G_ = 3.40, 95% CI = 1.46 – 7.94; *p* = 0.007) and (45% vs 14.7%; OR_G_ = 4.72, 95% CI = 2.35 – 9.46; *p* < 0.0001) respectively.

Conversely, the allele and genotype (TC and CC) frequencies of the rs1156361/C variant were similar in the two groups of patients divided according to disease severity (**Table 2**).

**Table 2:** Allele and Genotype distribution of rs35044562 and rs1156361 in SARS-CoV-2 patients.

Allele and Genotype distribution						
Gene	SNP	Asymptomatic, pauci-symptomatic, moderate (n = 318)			Severe (n = 40)	
*LZTFL1*	rs35044562 (A>G) (n = 358)	Allele	(%)	$X_{HWE}^{2}$ 0.0007, *p* = 0.979	(%)	$X_{HWE}^{2}$ 3.372, *p* = 0.066
		A	92.1		77.5	
		G	7.9		22.5	
		Genotype	(%)		(%)	
		AA	84.9		55	
		AG	14.5		45	
		GG	0.6		0	
*OAS3*	rs1156361 (T>C) (n = 358)	Allele	(%)	$X_{HWE}^{2}$ 0.019 *p* = 0.890	(%)	$X_{HWE}^{2}$ 0.026 *p* = 0.872
		T	35.2		32.5	
		C	64.8		67.5	
		Genotype	(%)		(%)	
		TT	12.6		10	
		TC	45.3		45	
		CC	42.1		45	

$X_{HWE}^{2}$ =Hardy-Weinberg equilibrium Chi square value; p = Hardy-Weinberg equilibrium p value.

The frequencies of the two allelic variants analyzed, rs35044562 and rs1156361, were consistent with the HWE in both groups of patients divided according to the severity of the clinical manifestations: X^2^HWE = 0.0007, *p* = 0.979 for rs35044562 and X^2^HWE = 0.019, *p* = 0.890 for rs1156361 in the first group of patients; X^2^HWE = 3.372, *p* = 0.066 for rs35044562 and X^2^HWE = 0.026, *p* = 0.872 for rs1156361 in the second group of severely ill patients.

The distribution of the alleles and genotypes observed for the two SNPs (rs35044562 and rs1156361*)* were also determined in the four distinct groups of patients, stratified according to their disease severity: asymptomatic (n = 120), pauci-symptomatic (n = 90), moderate (n = 108) and severe (n = 40). The statistically significant differences observed for rs35044562 are reported in **Table 3**.

**Table 3:** Association of severe COVID-19 with *LZTFL1* polymorphisms in an allele model.

rs35044562genotype							
COVID-19 Clinical Manifestations	AA	AG + GG	χ2	d.f.	*p*	OR (95% CI)	
Severe (n = 40)	22	18					
vs							
Asymptomatic (n = 120)	104	16	17.98	1	0.000	5.32	(2.53 - 12.01)
Pauci-symptomatic (n = 90)	80	10	18.82	1	0.000	6.55	(2.65 - 16.19)
Moderate (n = 108)	86	22	8.978	1	0.003	3.20	(1.47 - 6.97)
Asymptomatic, Pauci-symptomatic, Moderate (n = 318)	270	48	21.13	1	0.000	4.60	(2.29 - 9.22)

X^2^ = Chi square; d.f., degrees of freedom; p, p value; OR, Odds ratio (s), CI, confidence intervals.

The G allele of rs35044562 significantly increased the risk of developing severe COVID-19. This allele frequency was significantly reduced in the asymptomatic (13.3% vs 45%; OR_G_ = 5.32, 95% CI = 2.53 – 12.01; *p*< 0.0001) and pauci-symptomatic (11.1% vs 45%; OR_G_ = 6.55, 95% CI = 2.65 – 16.19; *p*< 0.0001) groups of patients and in the third group of moderately ill patients (20.4% vs 45%; OR_G_ = 3.20, 95% CI = 1.47 – 6.97; *p* = 0.003).

When these three groups were considered together for comparison with the more severe form of COVID-19, clear evidence of association emerged (17.8% vs 45%; OR_G_ = 4.60, 95% CI = 2.29 – 9.22; *p*< 0.0001).

The protective effect against severe COVID-19 reported for the rs1156361 variant was also evaluated. However, we did not find any significative differences in the genetic distribution of this SNP among the four groups of patients (**Table S2**). Furthermore, we found no correlation for the contemporary presence of both the risk (rs35044562) and protective (rs1156361) SNPs.

### Correlation Between rs35044562, rs1156361 and Immunogenetic Factors in the Different Clinical Forms of SARS-CoV-2 Infection

The next step was to try to understand why the rs35044562 high-risk variant, with such a high frequency in the Sardinian population, did not translate into an elevated incidence of severe clinical cases and mortality. We hypothesized that in Sardinia specific characteristics of the HLA system and/or other immunogenic factors could have a role in counteracting the negative effect exerted by the Neanderthal risk allele.

First of all, we examined the most frequent extended HLA haplotypes within the Sardinian population, and it readily became apparent that none of the 358 COVID-19 patients carried the extended haplotype HLA A*02:05, B*58:01, C*07:01, DRB1*03:01, which confirms the protective effect found for this extended haplotype in a previous study of 182 COVID-19 patients (12).

**Figure 1:**
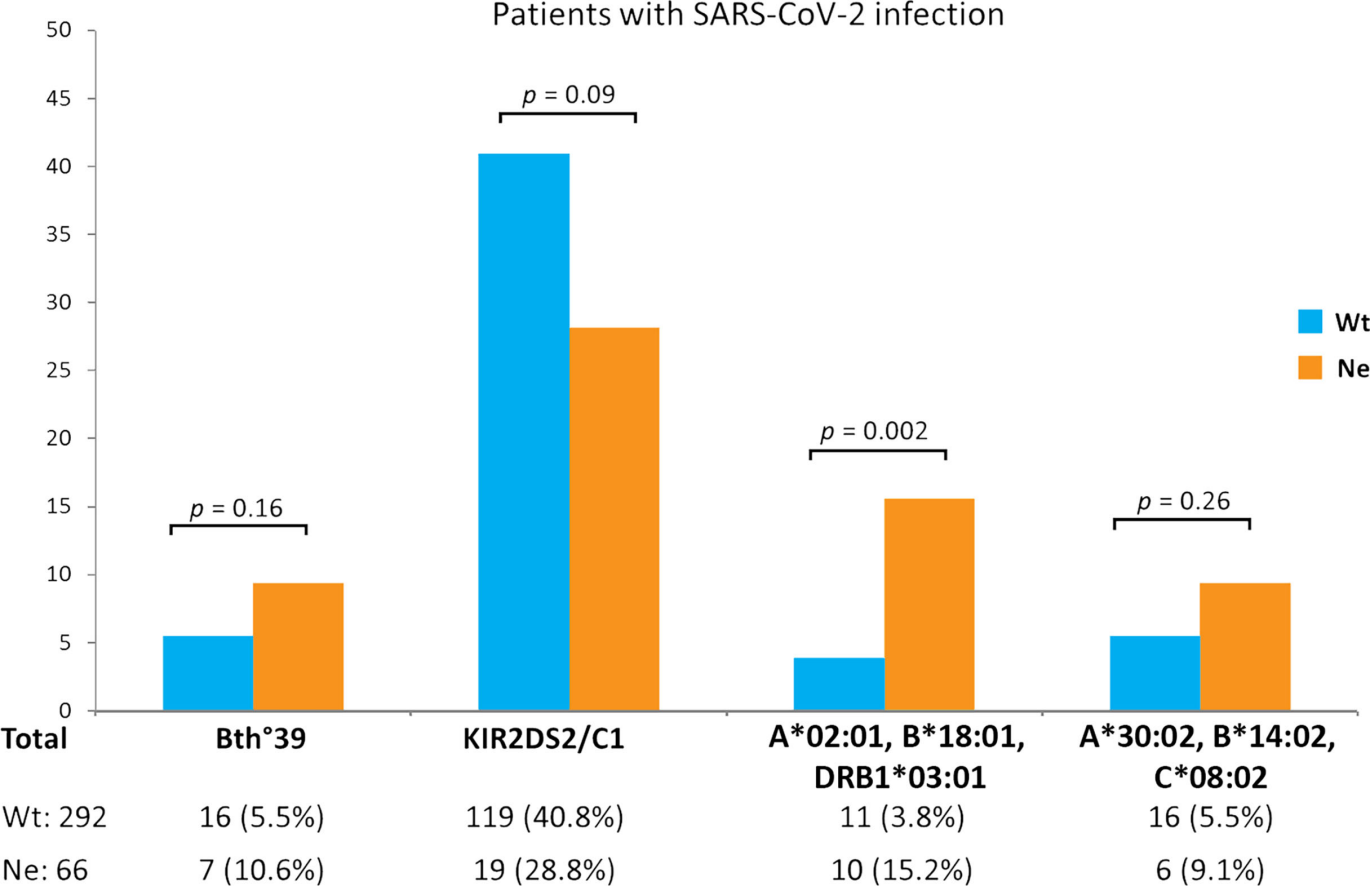
Evaluation of differences in immunogenetic factors associated to SARS-CoV-2 infection in patients carrying either the Wild Type allele variant (homozygous for rs35044562/A = Wt) or Neanderthal allele variant (presence of rs35044562/G = Ne) of the leucine zipper transcription factor-like 1 (*LZTFL1*) gene: the β^0^-39 (C>T) mutation of the beta globin chain (thalassemia trait), the *KIR2DS2*/HLA-C1 functional unit and the HLA-A*02:01, B*18:01, DRB1*03:01 or HLA-A*30:02, B*14:02, C*08:02 three-loci extended haplotypes. P-values (*p*) were calculated using the two-tailed Fisher exact test.

Among the remaining extended HLA haplotypes considered for correlation to the Neanderthal high-risk variant, the greatest difference between the two groups of SARS-CoV-2 patients was observed for the HLA-A*02:01, B*18:01, DRB1*03:01 three-loci haplotype which was significantly more frequent in the Neanderthal allele variant group (presence of rs35044562/G = Ne) than in the Wild Type allele variant group (homozygous for rs35044562/A = Wt) [15.2% vs 3.8%, OR 4.6 (95% CI 1.8 - 11.3), *p* = 0.002]. **Figure 1** shows the frequencies of the main immunogenetic factors considered in the two groups of patients stratified according to the presence or absence of the Neanderthal high-risk variant (rs35044562/G = Ne) or Wild Type variant (rs35044562/A = Wt).

**Figure 2:**
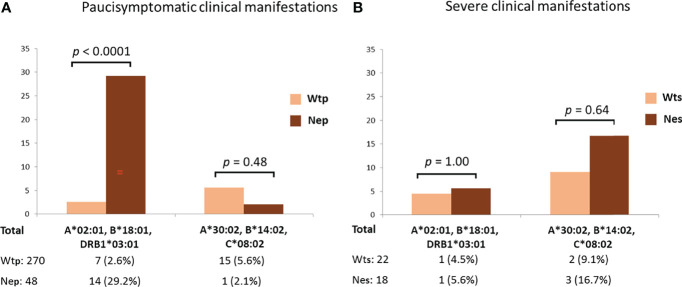
Frequencies of the HLA-A*02:01, B*18:01, DRB*03:01 or HLA-A*30:02, B*14:02, C*08:02 three-loci extended haplotypes in asymptomatic, pauci-symptomatic, moderate **(A)** and severely ill **(B)** patients subdivided according to the presence of the Wild Type allele variant (homozygous for rs35044562/A = Wt) or Neanderthal allele variant (homozygous or heterozygous for rs35044562/G = Ne) of the leucine zipper transcription factor-like 1 (*LZTFL1*) gene. P-values (*p*) were calculated using the two-tailed Fisher exact test. Wtp = pauci-symptomatic patients carrying the rs35044562/A variant allele; Nep = pauci-symptomatic patients carrying the rs35044562/G variant allele; Wts = severely ill patients with the rs35044562/A allelic variant; Nes = severely ill patients with the rs35044562/G variant allele.

The protective effect of the HLA-A*02:01, B*18:01, DRB1*03:01 haplotype became even more apparent when distinguishing between patients with asymptomatic/pauci-symptomatic/moderate and the severe forms of COVID-19 (**Figure 2**). In fact, pauci-symptomatic patients (Nep group) carrying the rs35044562/G variant allele had a very high frequency of HLA-A*02:01, B*18:01, DRB1*03:01 (29.2%) compared to pauci-symptomatic patients (Wtp group) carrying the rs35044562/A variant allele (2.6%) [OR 15.47 (95% CI 5.8 – 41.0), *p* < 0.0001].

No significant differences were observed for the HLA-A*30:02, B*14:02, C*08:02 haplotype between the two groups of patients divided according to the presence of the Wt (homozygous for rs35044562/A) or Ne (presence of rs35044562/G) variant of the *LZTFL1* gene [5.6% Wtp vs 2.1% Nep, OR 2.7 (95% CI 0.4 – 21.4), *p* = 0.48 and 9.1% Wts (severely ill patients with the rs35044562/A allelic variant) vs 16.7% Nes (severely ill patients with the rs35044562/G variant allele), OR 2.0 (95% CI 0.3 – 13.5), *p* = 0.64 respectively].

It is interesting to note that although not reaching statistical significance, the frequency of the *KIR2DS2*/HLA-C1 functional unit was reduced in the Ne group in comparison to the Wt group of patients [28.8% vs 40.8%, OR 1.7 (95% CI 0.9 – 3.0), *p* = 0.09].

## Discussion

A myriad of studies has been conducted to investigate the distribution, spread and rate of SARS-CoV-2 infection throughout the world, revealing differences in pandemic mitigation strategies between countries, populations, and regions that all urgently need to be addressed (41–44). Meanwhile, a multitude of factors contributing to the transmission of infection and disease outcomes continue to emerge. Many of these factors are likely influenced by genetic variations among populations (45, 46). The differences observed between Italian regions for the rate of SARS-CoV-2 transmission cannot simply be explained by geographical variations such as a low population density. Within this context, Sardinia has a lower infection rate than other Italian regions comparable for population density as well as the lowest incidence of hospital admissions for severe COVID 19 infection and mortality (**Table S3**) (8, 9). It would therefore seem that Sardinia offers a particularly useful population model from which to extrapolate some of the reasons behind the spread of infection. While insularity in certain situations may give rise to a population that is less susceptible to infection, it does not sufficiently explain why infection rates were so low on Sardinia throughout the pandemic. Despite the influx of millions of tourists during the summers of 2020 and 2021, there were fewer infections than in other regions (47).

The peculiar structure of the Sardinian population could account for one of the protective mechanisms. In fact, it has been shown that certain immunogenetic characteristics of the Sardinian population confer protection against the risk of severe COVID-19 infection. In particular, some conserved extended haplotypes of the major histocompatibility complex (MHC) and the presence of the killer cell immunoglobulin-like receptor (KIR) gene *KIR2DS2* in combination with HLA-C alleles of the C1 group (HLA-C1) significantly counteract the risk of developing severe clinical manifestations in Sardinian COVID-19 patients (12, 22).

In Sardinia, the frequency of the rs35044562 Neanderthal variant deviates significantly from that observed in Europe and the Middle East, where homo neanderthalensis lived following the phase of greatest expansion of the species occurring approximately 200.000 – 100.000 years ago. In comparison to other Italian regions (**Table S4**), Sardinia appears to be the one with the highest frequency of this polymorphism. The significant deviation from European and Tuscany frequencies may possibly be explained by an important founder event and the subsequent Neolithic expansion in some areas of Sardinia (48). Conversely, we did not find significant differences for the distribution of the rs1156361 protective variant in Sardinia compared to other ethnicities (**Table S5**).

The main endpoint of this study was to find out why Sardinia has one of the lowest incidences of hospitalization and related mortality in spite of a very high frequency of the Neanderthal variant (rs35044562) considered to be a major risk factor for a severe SARS-CoV-2 disease course.

A protective factor could be the β^0^-39 (C>T) variant of the beta globin gene (beta–thalassaemia trait), present in about 8-10% of the Sardinian population (49). However, the frequencies of the β^0^-39 variant were similar among SARS-CoV-2 infected patients, regardless of the presence of the Ne or Wt variants (*p* = 0.16).

Another protective factor against severe SARS-CoV-2 infection in Sardinia is represented by the presence of the functional unit *KIR2DS2* in combination with the HLA-C alleles of the C1 group (*KIR2DS2*/HLA-C1) (9, 22). Natural killer (NK) cells are vital to both anti-viral and anti-tumor immune response mechanisms. A plurality of NK cell functions is mediated by an array of inhibitory and activating killer-cell immunoglobulin-like receptors (KIR) expressed on the NK cell surface which predominantly bind to HLA-C ligands on target cells (50).

We could therefore plausibly expect the *KIR2DS2*/HLA-C1 functional unit to override the risk posed by the Neanderthal variant but once again no statistically significant differences (*p* = 0.09) were observed between patients carrying the Ne variant and those with the Wt variant (**Figure 1**).

Finally, we considered the HLA haplotypes present in all groups and found that the three-loci extended HLA haplotype HLA-A*02:01, B*18:01, DRB1*03:01 is associated with asymptomatic or pauci-symptomatic disease, while the HLA-A*30, B*14, C*08 haplotype is more frequently observed in patients with a severe or even fatal disease course (12). In fact, a marked difference was observed between the two groups of SARS-CoV-2 patients for the HLA-A*02:01, B*18:01, DRB1*03:01 three-loci haplotype which was significantly more frequent (15.2% vs 3.8%, *p* = 0.002) in the Ne group than in the Wt group (**Figure 1**). This three-loci extended haplotype has a relatively high frequency in the Sardinian population (51, 52) and is associated with asymptomatic or pauci-symptomatic disease (12).

The protective effect of the HLA-A*02:01, B*18:01, DRB1*03:01 extended haplotype in the Ne group of patients became even more apparent when distinguishing between patients with asymptomatic/pauci-symptomatic/moderate (Nep) or severe (Nes) forms of COVID-19 (**Figure 2**). Indeed, the Nep group had a very high frequency of HLA-A*02:01, B*18:01, DRB1*03:01 compared to the pauci-symptomatic patients of the Wtp group (29.2% vs 2.6%, p < 0.0001). Therefore, the high frequency of this haplotype in patients with the Neanderthal polymorphism (15.2%), particularly pauci-symptomatic patients (29.2%), supports the hypothesis that this specific HLA profile may confer a certain degree of protection against severe SARS-CoV-2 infection, even when challenged by the presence of this high-risk Neanderthal variant.

Despite the recent increases in cases attributable to the rapid spread of the Omicron variant, Sars-CoV-2 transmissibility indexes and intensive care admission rates in Sardinia continue to be among the lowest registered among the different Italian regions. Sardinia, with its low rate of hospital admissions and ICU occupancy levels, has also been confirmed as one of the areas in Europe with the lowest incidence of severe cases of SARS-CoV-2 infection (9). Hence, the protection conferred by the HLA-A*02:01, B*18:01, DRB1*03:01 three-loci extended haplotype against severe SARS-Cov-2 in the Sardinian population seems to efficiently contrast the high risk of a severe and devastating clinical course of the infection predicted for carriers of the Neanderthal haplotype.

Interestingly, two alleles (HLA-A*02:01, B*18:01) of this haplotype are also part of the HLA haplotype, HLA-A*02.01g-B*18.01g-C*07.01g-DRB1*11.04g, which in Italy has a regional distribution inversely correlated with both the incidence and mortality of COVID-19 (51). Although this protective haplotype is the second most prevalent haplotype in the Italian population (51), in Sardinia it is one of the haplotypes with the lowest frequency (19, 52). In fact, it was not present in our study population.

In our study the protection conferred by the HLA-A*02:01, B*18:01, DRB1*03:01 three-loci extended haplotype clearly outweighed the risk conferred by the high-risk variant inherited from Neanderthals. This finding strongly suggests that the balance between risk and protective immunogenetic factors is extremely important to the evolution of COVID-19.

There is an ongoing global hunt for people genetically resistant to SARS-CoV-2 infection in the hope of identifying genes or other immunogenetic factors that can be used for the development of drugs that can protect people and prevent them from transmitting the disease. In the search for resistance to HIV infection, researchers were able to identify a rare mutation capable of disabling the CCR5 receptor on white blood cells preventing the virus from infiltrating the immune system *via* this access point (53). In the same way, some people may possess specific haplotypes and/or variants in genes that could stop SARS-CoV-2 from entering cells or break down viral RNA within cells or even inhibit its replication and repackaging into new viral particles (19, 54–56).

The Island population of Sardinian has a relatively low level of genetic heterogeneity and has been considered by many researchers as an ideal ground for the study of genetic variants that may be linked to disease. However, we advocate the combined input of researchers worldwide to further uncover the immunogenetic mechanisms underlying inborn resistance to SARS-CoV-2 infection. A better understanding of these mechanisms may well turn out to be the biggest advantage in the race for the development of efficient drugs or vaccines against COVID-19.

## Data Availability Statement

The data presented in the study are deposited in the Bioproject repository, accession number PRJNA823895.

## Ethics Statement

The studies involving human participants were reviewed and approved by Ethics Committee of the University of Cagliari, Italy. The patients/participants provided their written informed consent to participate in this study.

## Author Contributions

Each author listed has made a direct, substantial, and intellectual contribution to the work, and approved it for publication.

## Conflict of Interest

The authors declare that the research was conducted in the absence of any commercial or financial relationships that could be construed as a potential conflict of interest.

## Publisher’s Note

All claims expressed in this article are solely those of the authors and do not necessarily represent those of their affiliated organizations, or those of the publisher, the editors and the reviewers. Any product that may be evaluated in this article, or claim that may be made by its manufacturer, is not guaranteed or endorsed by the publisher.
