# Supplementary material for: A Protective HLA Extended Haplotype Outweighs the Major COVID-19 Risk Factor Inherited From Neanderthals in the Sardinian Population
Source: Front Immunol. 2022 Apr 19;13:891147. doi: 10.3389/fimmu.2022.891147 (PMC9063452; doi:10.3389/fimmu.2022.891147)
Supplement: Supplementary file 1 [file DataSheet_1.docx]

Supplementary Material

**Table S1. RefSNP ID position and primer data.**

| RefSNP ID | Position (GRCh38) | Gene | Primer name | Sequence | Length | Tm | GC% |
| --- | --- | --- | --- | --- | --- | --- | --- |
| rs73064425 | chr3:45859597 | *LZTFL1* | rs73064425_For | GTCAGTAGTTCAGGACCAGCC | 21 | 60 | 57 |
|  |  |  | rs73064425_Rev | CTAGCCATGGTGCCTGACTATT | 22 | 60 | 50 |
| rs35044562 | chr3:45867532 | *LZTFL1* | rs35044562_For | CGTTGTTGTTTTGAGACAGGGT | 22 | 60 | 45,5 |
|  |  |  | rs35044562_Rev | AAGGACAGTTCTGGCACAAGAA | 22 | 60 | 45,5 |
| rs34326463 | chr:3:45899651 | *LZTFL1* | rs34326463_For | TCAGAGTAAGGAAGAGCAACACA | 23 | 59 | 43,5 |
|  |  |  | rs34326463_Rev | CACATATGCCTCCCACTCCTTA | 22 | 59 | 50 |
| rs67959919 | chr3:45830416 | *LZTFL1* | rs67959919_For | ATTAGCAAAAGCCCAGTGAAGC | 22 | 60 | 45,5 |
|  |  |  | rs67959919_Rev | CCCCTCTCACTTTGACATCCAA | 22 | 60 | 50 |
| rs1156361 | chr12:112938178 | *OAS3* | rs1156361_For | ACAGCGAGGTAGGACTTCTCC | 21 | 60 | 57 |
|  |  |  | rs1156361_Rev | CCAGCTACGACTTCCTCTCCT | 21 | 60 | 57 |
| rs11549407 | chr11: 5226774 | *HBB* | rs11549407 For  rs11549407 Rev | AGAAACTGGGCATGTGGAG  AGAAAACATCAAGCGTCCC | 19  19 | 52  47 | 57  57 |

Tm = Primer melting temperature
GC% = Guanine and Cytosine content percentage

**Table S2*.* Odds ratio (ORs) and 95% confidence intervals (CIs) for association of severe COVID-19 with *OAS3*polymorphisms in an allele model.**

| rs1156361 genotype | | | | | | |
| --- | --- | --- | --- | --- | --- | --- |
| COVID-19 Clinical Manifestations | TT | TC+CC | χ2 | d.f. | *p* | OR (95% CI) |
| Asymptomatic (n= 120) | 16 | 104 |  |  |  |  |
| vs |  |  |  |  |  |  |
| Pauci-symptomatic (n= 90) | 12 | 78 | 0.000 | 1 | 1.00 | 1.00 (0.49 - 2.23) |
| Moderate (n= 108) | 12 | 96 | 0.261 | 1 | 0.61 | 0.81 (0.37 - 1.81) |
| Severe (n= 40) | 4 | 36 | 0.305 | 1 | 0.58 | 0.72 (0.23 - 2.30) |

^χ 2^ = Chi square; d.f. degrees of freedom; *p* = p value

**Table S3. Ratio of people infected by SARS-CoV-2 in different Italian regions.**

| **Regions** | **Resident Population^*^** | **Population density**  **(inhabitants / km²)*** | **COVID-19 cases per million people**** | **COVID-19 death per million people**** |
| --- | --- | --- | --- | --- |
| Sardinia | 1 640 000 | 66 | 72 561 | 1 104 |
| Abruzzo | 1 312 000 | 118 | 150 808 | 2 108 |
| Basilicata^#^ | 562 869 | 54 | 103 264 | 1 190 |
| Calabria^#^ | 1 947 000 | 122 | 82 952 | 933 |
| Campania | 5 802 000 | 411 | 167 037 | 1 545 |
| Emilia-Romagna | 4 459 000 | 198 | 215 645 | 3 330 |
| Friuli-Venezia Giulia | 1 215 000 | 151 | 202 116 | 3 641 |
| Lazio | 5 879 000 | 333 | 137 928 | 1 650 |
| Liguria | 1 551 000 | 280 | 175 087 | 3 114 |
| Lombardy | 10 060 000 | 418 | 200 418 | 3 646 |
| Marche | 1 525 000 | 159 | 152 235 | 2 207 |
| Molise^#^ | 305 617 | 66 | 90 417 | 1 711 |
| Piedmont | 4 356 000 | 168 | 187 204 | 2 870 |
| Puglia | 4 029 000 | 201 | 139 406 | 1 775 |
| Sicily | 5 000 000 | 187 | 116 575 | 1 657 |
| South Tyrol | 520 891 | 72 | 136 975 | 2 231 |
| Tuscany | 3 730 000 | 161 | 183 305 | 2 168 |
| Trentino | 538 223 | 77 | 81 964 | 2 499 |
| Umbria | 882 015 | 102 | 164 507 | 1 816 |
| Aosta Valley | 125 666 | 38 | 218 953 | 3 979 |
| Veneto | 4 906 000 | 265 | 216 860 | 2 652 |
| Italy | 59 550 000 | 196 | 171 485 | 2 424 |

* Eurostat source (3), ** Data update in December 2021 from the Italian Ministry of Health (4), # Partial data

**Table S4. Frequency of the Neanderthal allele variant (rs35044562/ G) allele variant in different populations.**

| **Population** | **Ne Allele Count *** | **Ne Allele Number**** | **Number of Ne Homozygotes** | **Ne Allele Frequency** |
| --- | --- | --- | --- | --- |
| African/African-American^1^ | 99 | 8 708 | 0 | 0 .01137 |
| Ashkenazi Jewish^1^ | 37 | 290 | 2 | 0 .12760 |
| East Asian^1^ | 1 | 1 556 | 0 | 0 .00064 |
| European (Finnish) ^1^ | 251 | 3 476 | 6 | 0 .07221 |
| European (non-Finnish) ^1^ | 1 229 | 15 386 | 57 | 0 .07988 |
| Latino/Admixed American^1^ | 43 | 848 | 2 | 0 .05071 |
| Tuscany^2^ | 21 | 214 | 0 | 0 .09813 |
| Sardinia | 145 | 1344 | 5 | 0 .10790 |

^1^gnomAD (2021). Available at: https://gnomad.broadinstitute.org/ (Accessed February 11, 2022).

^2^Data source ensembl.org (doi: 10.1093/nar/gkaa942) Accessed February 11, 2022.

Ne = Neanderthal allele variant **(**rs35044562 A>G).

*Alternate allele count in high quality genotypes,**total number of called high quality genotypes

**Table S5. Frequency of the rs1156361/C allele variant in different populations.**

| **Population** | **Allele Count*** | **Allele Number**** | **Number of Homozygotes** | **Allele Frequency** |
| --- | --- | --- | --- | --- |
| African/African-American^1^ | 8146 | 8708 | 3809 | 0.9355 |
| Ashkenazi Jewish^1^ | 164 | 290 | 51 | 0.5655 |
| East Asian^1^ | 1225 | 1552 | 481 | 0.7893 |
| European (Finnish) ^1^ | 2541 | 3474 | 923 | 0.7314 |
| European (non-Finnish) ^1^ | 10210 | 15404 | 3376 | 0.6628 |
| Latino/Admixed American^1^ | 651 | 844 | 247 | 0.7713 |
| Tuscany^2^ | 120 | 214 | 33 | 0.5607 |
| Sardinia | 878 | 1344 | 288 | 0.6533 |

^1^gnomAD (2021). Available at: https://gnomad.broadinstitute.org/ (Accessed February 11, 2022).

^2^Data source ensembl.org (doi: 10.1093/nar/gkaa942) Accessed February 11, 2022.

*Alternate allele count in high quality genotypes, **Total number of called high quality genotypes
